# End Malaria Faster: Taking Lifesaving Tools Beyond “Access” to “Reach” All People in Need

**DOI:** 10.9745/GHSP-D-22-00118

**Published:** 2022-04-28

**Authors:** Courtney Emerson, Jed Meline, Anne Linn, Julie Wallace, Bryan K. Kapella, Meera Venkatesan, Richard Steketee

**Affiliations:** a U.S. President's Malaria Initiative, U.S. Centers for Disease Control and Prevention, Atlanta, GA, USA.; b U.S. President's Malaria Initiative, United States Agency for International Development, Washington, DC, USA.

## Abstract

To “reach the unreached” with preventive and curative malaria services, we must know which individuals and communities remain unreached and then bring tailored services from the clinic to the community and home.

## Key Messages

- To effectively address malaria control and elimination worldwide, we must endeavor to “reach the unreached,” to deliver malaria services from the clinic to the community and home.
- **Reach** moves beyond **access** and requires that we have the data to know who are unreached, where they are located, and how to ensure they receive malaria services.
- **Reach** can only be achieved with community health workers that are adequately supported and equipped to diagnose and treat malaria in every person in their communities regardless of age.
- **Reach** incorporates equity and responsibility for service delivery more expansively.

## BACKGROUND

Malaria causes hundreds of millions of infections and kills more than half a million people each year.^1^ The U.S. President's Malaria Initiative (PMI) works in close partnership with National Malaria Control Programs (NMCPs) and other partners in 24 malaria-endemic African nations and 3 programs in the Greater Mekong subregion to change that.^2^ PMI recently released its 2021–2026 Strategy *End Malaria Faster.*^3^ The Global Fund recently released its new Strategy 2023–2028, which includes a malaria-specific technical strategy for the first time.^4^ The World Health Assembly also adopted the Update to the Global Technical Strategy and Targets for Malaria 2016–2030 in May 2021.^5^ Each of these strategies aims to support NMCPs and their partners to deliver lifesaving interventions—such as insecticide-treated nets (ITNs) and indoor residual sprays (IRS) on house walls that kill malaria parasite-carrying mosquitoes, preventive use of antimalarial drugs, and tests and medicines to diagnose and treat malaria. PMI, along with these other key malaria actors, also invests in strengthening the capacity of health workers, laboratories, supply chains, surveillance, and other health system pillars to control and eliminate malaria, save lives, and strengthen global health security.^6^

Historic progress against malaria is threatened. The World Health Organization (WHO) estimates there were 241 million malaria cases and 627,000 deaths worldwide in 2020—figures that had steadily declined since 2000 but began to stagnate in 2015.^1^ Then, with the advent of the coronavirus disease (COVID-19) pandemic, malaria morbidity and mortality increased for the first time in this century. Efforts to achieve ambitious global targets to reduce malaria have dramatically fallen short. The resource gap grows each year with increasing populations in endemic areas; WHO has noted that 2020 funding reached US$3.3 billion against an estimated need of US$6.8 billion.^1^ New threats are widening this divide. The impacts of COVID-19 on communities, the health workforce, supply chains, and health systems may have further set malaria progress back by years.^7^ Increasing antimalarial drug and insecticide resistance and growing conflict and violence in malaria-affected communities pose major challenges to progress.^1^ A reversal in progress against malaria could have dire consequences, resulting in hundreds of thousands of additional deaths, potentially increasing the risk of outbreaks and drug resistance, undermining economies, increasing poverty, and weakening global health security.^8^

Despite strong progress in scaling up malaria interventions across malaria-endemic sub-Saharan Africa, by 2020, 35% of homes did not have at least 1 ITN, and care was not sought for more than 30% of children with fever.^1^ These individuals often represent families experiencing poverty and living in rural areas, but significant numbers also live near health facilities. Where care is available, critical gaps persist in the quality of services including malaria testing and treatment practices, and in the deployment of optimal mosquito nets to address insecticide resistance.^9^ Though we have existing tools that are highly effective, coverage remains suboptimal. There is a need to focus on ways to ensure that preventive interventions like ITNs and seasonal malaria chemoprevention (SMC), as well as prompt and effective malaria case management services, reach families who are not yet using these effective interventions.

There is a need to focus on ways to ensure that malaria services reach families who are not yet using these effective interventions.

The global malaria community has been engaged in dialogue regarding how to return to a course of progress, even “Rethinking Malaria.”^10^ The new PMI strategy calls for a focus to “Reach the Unreached.” Strategic investments in community health systems and surveillance can better extend prevention and care to the unreached and strengthen pandemic preparedness and response. While there is a need for new tools for malaria control^11^ and the promise of new malaria vaccines,^12^^,^^13^ existing tools, including ITNs, IRS, preventive treatment, and case management, have not yet achieved their full potential due to the number of families and individuals not yet being reached by these lifesaving interventions.^14^^,^^15^

## DEFINING “REACHING THE UNREACHED”

**Access** is defined in general terms as “permission, liberty, or ability to enter, approach, or pass to and from a place or the freedom or ability to obtain something.” By contrast, **reach** is defined as “to extend to, to come to, to communicate with” and denotes a much more active, expansive approach with close engagement and accompaniment.^16^^,^^17^ Within a global health context, care is accessible if it is available when and where people need it.^18^

Reaching the unreached is an active extension of accessible health care. Reach incorporates the utilization of prevention and care services, beyond availability, and is the outcome of working to achieve, sustain, and tailor deployment and uptake of high-quality, proven interventions with a focus on people who have been underserved. Reach builds on access and requires communities to have appropriate knowledge about care seeking and using preventive and treatment services, which necessitates identifying and addressing various barriers and facilitators experienced by specific segments of the population that underuse priority interventions. Reach addresses the critical needs of all populations in and outside of communities (e.g., migrant and mobile populations) and requires overcoming economic and a myriad of other barriers to care. Reach has implications well beyond access to ensure that individuals and communities both have and use their ITN; get tested for their febrile illness; obtain the diagnostic result, including the needed dose of medication; and receive their preventive antimalarial treatment (e.g., intermittent preventive treatment during pregnancy [IPTp] or SMC). In addition, reaching the unreached can sometimes refer to reaching adults in a community where government- or donor-supported services target only young children.

Previous studies have shown the importance of strong health systems to deliver preventative and curative malaria services,^19^^,^^20^ and this continues to require focus. However, reach also emphasizes the importance of the quality of prevention, care, and treatment, recognizing that people receiving poor-quality services are not “effectively covered” and are essentially unreached.^9^ While there are few specific scientific references to reaching the unreached,^21^^–^^23^ efforts to extend services to populations in need are evident in many programs, and evidence exists from large-scale intervention trials that the action of taking existing recommended interventions to high coverage in populations can have a marked impact in preventing infection, illness, and death.^24^^,^^25^ By using the term “reach” instead of “access,” the dialogue moves perceptibly further toward impact.

By using the term “reach” instead of “access,” the dialogue moves perceptibly further toward impact.

Community characteristics can determine the best strategies to reach families and individuals. Population characteristics such as rural/urban, minority, migratory or mobile, and internally displaced/conflict-affected may lead to distinct and varying local needs and delivery requirements. As noted in the WHO Strategy, there is a need to address health disparities and inequities to respond to populations experiencing disadvantage, discrimination, or exclusion.^14^

Malaria services can be thought of as a package of high-quality tools or interventions that can be delivered to all of those in need when (1) commodities are appropriately resourced, procured, distributed, and monitored; (2) personnel are sufficiently trained and compensated; and (3) beneficiaries understand and have the desire and means to take advantage of each intervention. There is diversity in the strategies available to reach susceptible populations with these services. For example, with vector control tools, ITNs need to be in the home and used to cover sleeping spaces; IRS must be taken to every appropriately targeted house and sprayed on walls; diagnostics and artemisinin-based combination therapies for confirmed malaria must be delivered at the home, health post, or clinic and used for the child with suspected malaria. For a child with fever to get tested promptly, families and providers must connect. IPTp must be actively delivered to pregnant women, and young children in areas of highly seasonal transmission must be reached repeatedly in their homes or communities with SMC. Social and behavior change messaging and interventions are a critical component of an effective malaria service package by helping ensure access is converted into reach and services are used.^26^ Reach requires a skilled and supplied workforce to deliver tools and an informed community to accept/make use of them. Only then will the tools, existing or new, be taken to those in need in a way that truly reaches that population.

## KNOWING WHO IS UNREACHED AND HOW TO REACH THEM

Understanding **reached** and **unreached** requires measurement and quantification of those who do and those who do not use the necessary interventions (e.g., with ITN ownership of 70%, who are the 30% with no ITN?). In fact, with our current measurements of malaria intervention coverage, we are often (but not always) measuring the scale of population reach. Unreached populations are different in each country and locality. To reach them requires a deeper understanding of the population to determine who is unreached, where they can be found, what characteristics they have, and how we can ensure the necessary prevention or care services are utilized. Local knowledge is critical. This means that people in communities help identify the unreached and identify options to establish reach. Data and information systems can then be designed and made available locally to help communities further plan and map their actions.

Local knowledge is critical: people in communities can help identify the unreached and identify options to establish reach.

Countries have made significant progress in strengthening data systems. Many countries have adopted electronic health information systems, which have improved data collection and may help to reduce stockouts.^27^^,^^28^ Malaria surveillance is a key intervention component of the WHO Global Technical Strategy.^14^ Recent advances in mobile technology have enabled health workers in remote communities to communicate with supervisors, record and report data, receive on-the-spot virtual support, and even geo-map cases and prevention services. As the cost of such tools continues to fall and connectivity improves, they can be used on a larger scale to improve both the quality of services provided and the data for surveillance and planning.^29^

The common information systems may inherently miss the unreached. Routine health management information systems report only on people who are reached at a health facility or sometimes by a community health worker (CHW) and typically exclude those not accessing services in the public sector (also those using the private sector or military facilities). Surveys (e.g., Demographic and Health Surveys,^30^ Multiple Indicator Cluster Surveys,^31^ Malaria Indicator Surveys,^32^ and Malaria Behavior Surveys^33^) have provided important population-level information on intervention coverage, infection and anemia rates, and certain knowledge and behaviors. However, these surveys are point-in-time snapshots every few years, and sampling frameworks only allow rate estimates at national or regional levels, thereby offering limited local granularity. Hence, they can often miss mobile and otherwise unreached or undocumented populations. These current tools have important value, yet as we push to define the unreached, local efforts will benefit when they explore and ultimately adopt additional information opportunities to assure that the unreached can be identified and ultimately reached.

Newly available systems to collect district and local data can be used to identify unreached populations and measure progress in reaching them. Systems such as the Geo-Referenced Infrastructure and Demographic Data for Development incorporated into the AKROS Reveal Platform, the Center for International Earth Science Information Network, and Ecopia AI can combine satellite imagery and geographic location including data on population, settlements, infrastructure, high-risk environments, and boundaries to discover unreached places.^34^^–^^36^ As satellite data typically rely on census or vital registry systems for validation, foundational investments in better vital registry systems in countries are also required,^37^ noting that countries that do not have these, have only an approximation of the number of individuals in their population.^38^

Newly available systems to collect district and local data can be used to identify unreached populations.

Finding groups that have been economically or socially marginalized will require different types of information, potentially gathering knowledge and data from private or nongovernmental organizational providers that may not be well connected to existing health systems. In addition, internally displaced or conflict-affected people may require outreach to local organizations that serve them and that may not be integrated into district health information software reporting systems.^39^

Data collection and interpretation related to complex emergencies is often different from regular malaria programming and is a critical aspect of reaching the unreached in these situations.^40^

Ironically, the unreached can sometimes be hidden in a community where government or donor-supported services target only young children or set other “specific coverage” targets.^41^ Children aged 5 years and older, older adolescents, and adults can often serve as reservoirs for malaria, not getting sick immediately or only modestly. Adults who would quickly seek care for a febrile child might defer their own care, risking their welfare and perpetuating malaria transmission in their communities. In many cases, child-focused services might be efficiently expanded to reach adults as well.

The unreached can sometimes be hidden in a community where government or donor-supported services target only young children or set other “specific coverage” targets.

Improved vital registration for all members of a population allows for better program planning by local health leaders. Data advances will allow national programs to better track disease trends and intervention effectiveness, identify coverage gaps, monitor commodity stocks in more remote areas, and identify populations requiring more community health services and the workforce to deliver them—in other words, help reach the unreached.

## IMPLICATIONS OF REACH: TAILORED MALARIA CONTROL PROGRAMS

With differences in geography, climate, malaria seasons, mosquito characteristics, cultural and social norms, and human behavior, it is important to identify the most effective mix of interventions for each setting.^29^ To truly reach communities with the right services, it is critical to not only identify areas of greatest malaria burden, greatest need, or critical communities missed but also then ensure that the systems are strong enough to consistently deliver quality care and prevention services to those communities and households we are trying to reach. Fully reaching all susceptible people can lead to demonstrable reductions in malaria infection and burden and help address the stagnation in global progress against malaria.^24^

Countries can tailor the deployment of interventions and intervention packages subnationally so that the right tools are available in the right places at the right time. Evidence from operational research, evaluations, modeling, and other data can guide the selection of interventions and their combinations. For example, tailoring includes using local data to distribute mosquito nets effective against insecticide resistance preferentially or extending the length of chemoprevention campaigns in districts with a longer malaria season.^42^ In elimination settings, different interventions may be used to target foci of local transmission or to prevent importation of malaria through exposure to unique high-risk populations.^43^

## ENHANCING COMMUNITY HEALTH SYSTEMS TO REACH THE UNREACHED

Bringing care to people helps ensure that no one remains unreached by lifesaving tools and services. Malaria tools do not deliver themselves—health workers do.^44^ One proven approach for delivering malaria services closer to home is CHWs, who, in many countries, are engaged in campaign-style prevention interventions as well as in the delivery of routine case management services.^45^ CHWs have demonstrated the ability to provide testing and treatment for malaria, along with diarrhea, pneumonia, and other childhood diseases through an integrated community case management (iCCM) platform, a proven approach for reducing child mortality when sufficient commodities for non-malaria ailments are available.^46^

Malaria tools do not deliver themselves— health workers do.

In countries or specific areas where malaria case rates are at lower levels, national programs are implementing proactive community case management where CHWs conduct regular household visits to actively seek out cases of fever and provide appropriate case management—proactively reaching community members where they reside rather than waiting for care to be sought.^47^ CHWs also play an active role in case follow-up and investigation in elimination settings. In addition, evidence and experience show that community-centered interventions can influence social norms, foster an environment for the practice of better health behaviors, and increase demand for broader health services.^48^^,^^49^ Yet CHWs cannot deliver these services without investment in systems to support them, which includes relationships and processes to support health in communities and households outside but related to the formal health system.^18^

There are many examples of system challenges that can limit the impact of CHWs. Weak and under-resourced systems for supervision leave CHWs without the support and mentorship they need to work effectively. Health management information systems that do not integrate community-level data or even numbers of CHWs^50^ leave the true impact of CHWs unmeasured.^46^^,^^51^ Most CHWs are women, many are people experiencing poverty, and most are not paid. Women on the front lines, including many at the community level, subsidize more than US$1 trillion of health care globally with their unpaid labor,^52^ and WHO recommends that CHWs be paid commensurate with their work.^53^

Frequent stockouts of commodities prevent CHWs from offering care for malaria, but in many cases, even more so for the other childhood diseases covered in the iCCM platform.^54^^,^^55^ Despite efforts by country governments and development partners to scale up iCCM platforms, many countries have reported that supplies needed for the treatment of diarrhea, pneumonia, and malnutrition are not consistently funded or available at the community level. It is important to extend the collaboration with partners in child health to advocate at global, national, and local levels for the provision of both malaria and nonmalaria commodities^56^ together so CHWs can offer a truly integrated suite of lifesaving services and reach the unreached for the full spectrum of health needs.^57^ It is also critical that this work is owned and coordinated by government leaders.^58^

Investment in communities and community health systems using iCCM has the potential to yield returns against malaria and more broadly against preventable and treatable diseases.^46^^,^^57^^,^^59^ Strengthening community health systems, from the clinic to the community level, is a second focus area in PMI's strategy, homing in on the “5 Ss” of community health systems.^60^

These include a community health workforce that is:

- **Selected** in the best way to get the right people providing this service in all the communities of need
- **Skilled**, either at the outset or through training
- **Supplied** with the necessary tools and equipment
- **Supervised** in a supportive way that invokes quality
- **Salaried** so that they can earn a living wage and sustain their engagement

A critical next step is to scale up community-based models of care to all ages where appropriate and extend reach to find and serve the unreached with malaria prevention, testing, and treatment.^61^

## CONCLUSIONS

With the current availability of highly effective malaria interventions, we have reached many communities and homes and reduced infections, illness, and death. However, coverage and use of these tools are still far short of needed levels which can further contribute to inequities and injustices. To address the plateau in progress against malaria, the global malaria control community needs to ensure quality interventions reach the unreached. Programs will benefit from learning who the unreached are, where they are located, and how to best get the most effective malaria preventive and treatment services to them in their communities and homes. This requires better, timelier, and fully available data for communities. Malaria services must be of sufficient quality and presented in a way that results in good use. Stronger community health systems, involving CHWs, can provide prevention, diagnosis, and treatment of malaria for all ages and are central to successfully reaching the unreached. PMI has committed to reaching the unreached as a critical component of its new strategy and will work with partners to again drive the malaria morbidity and mortality curves down and end malaria faster.

## Notes

**Peer Reviewed**

**First published online:** April 20, 2022.
